# Refugee Children and English Language: Challenges From English Language Teachers’ Perspectives

**DOI:** 10.3389/fpsyg.2022.918734

**Published:** 2022-06-22

**Authors:** Haneen Abdaljaleel Alrawashdeh, Naciye Kunt

**Affiliations:** Department of Foreign Language Education, Eastern Mediterranean University, Famagusta, Turkey

**Keywords:** refugees, psychological challenges, linguistic challenges, English as a foreign language, constructivism

## Abstract

This study aimed to investigate the challenges refugee children face in learning the English language from teachers’ perspectives and the challenges of refugees’ English language teachers in Jordan. To achieve this aim, a quantitative approach was implemented using a questionnaire. The findings from this study suggest that refugee teachers’ acknowledge that they face challenges teaching refugees in terms of cultural competency, preparation, self-efficacy, and practices they implement, and refugee children face linguistic and psychological challenges in learning English. This study attempted to find out the relationship between teachers’ preparation and their cultural competency, self-efficacy, and practices they implement and then the relationship between refugees’ psychological needs and linguistic challenges, respectively, and teachers’ self-efficacy, cultural competency, and practices. Results revealed several significant relationships between challenges and presented them in a model.

## Introduction

Jordan has hosted a high number of refugees since it is a country in the middle of a zone with political conflicts and wars, and it is the most responsive country that hosts refugees among all the neighboring countries. Currently, millions of refugees from different countries live in Jordan. Jordan (UNHCR, 2019) has received refugees from different countries, around 60,000 Iraqis, 14,000 Yemenis, 6,100 Sudanese, and 1,700 from other countries, such as Somalia. Similar to neighboring countries, such as Lebanon and Turkey, Jordan is under immense pressure to intake the waves of refugees. Salehyan and Gleditsch (2006) showed that the high number of refugees increases the challenges in social, economic, and educational aspects in the host country.

Refugees pass through hard circumstances after and before refuge since they were taught by different educational systems and curricula in their homelands, some have different native languages and some used to learn different foreign languages in schools, e.g., some Syrian refugees used to learn the French language instead of English, and all of these stand as challenges for refugees and their teachers to progress in language learning and teaching since constructing new knowledge and developing in language learning rely on endogenous elements, internal schema, previous experiences, and knowledge of learners combined with sociocultural and psychological factors (Cole, 1990).

This study investigates the challenges of teaching English as a foreign language (EFL) to refugees and the challenges of refugees in learning EFL from English teachers’ perspectives; accordingly, it attempts to provide a broader view of the challenges related to EFL and refugees and to enrich the literature by explaining in-depth the relationship between these challenges and present them in a comprehensive model. Practically, it provides authentic information that helps in enhancing the practices used to teach EFL to refugees.

## Constructivism

The theory of constructivism claims that in the language learning process, learners construct new ideas and concepts according to their previous knowledge. Piaget (1980), the father of constructivism, argued that learners take new information and ideas and then shape them upon their understandings, previous knowledge, and experiences. The construction of individuals’ new knowledge emerges according to two components; accommodation and assimilation, in assimilation, individuals relate new experiences to old ones, and accommodation means reshaping the new experiences into the mental capacity that already existed. Ginsborg (1990) wrote that new information, thoughts, and concepts can have meaning only when it is related to individuals’ knowledge, and this represents schema. Schema theory is “every act of comprehension that involves individual’s pre-existed knowledge” (Anderson et al., 1977). For example, readers of such text develop their interpretation through combining information the text provides with the information a reader adds to that text (Widdowson in Grabe, 1988:56). Constructivism can clarify the challenges the refugees face because they build the new knowledge according to the old knowledge which was stored using different curricula, different systems, and sometimes different languages in their home countries.

## A Review of Related Literature

Teaching EFL is a common research issue associated with the discipline of applied linguistics. Some previous studies (Kanno and Varghese, 2010; Lertola and Mariotti, 2017; Sujito et al., 2019) have identified some challenges of EFLs in many contexts. Undoubtedly, learning the English language has many benefits, e.g., increasing the effective communication (Lertola and Mariotti, 2017), raising job opportunities (Kanno and Varghese, 2010), and increasing social interaction; hence, refugee children need to learn the English language for self-empowerment; however, recent studies have revealed significant educational difficulties that refugee children face (Palaiologou, 2007); one of these difficulties is learning English as a foreign language (Hulistijn, 2011; Gizatullina and Sibgatullina, 2018). Refugees require extra time and effort learning a foreign language according to many reasons, such as dealing with new teachers and interacting with new classmates (AlHariri, 2018), attending new schools (Popov and Sturesson, 2015), having linguistic issues as the unfamiliarity with the language (Riggs et al., 2012), and being in a new society (Burgoyne and Hull, 2007). Similar to Lee (2016), refugees face sociocultural challenges because they come from various backgrounds which in turn restrict them from acculturation with individuals in the target context and, accordingly, they are not confidently interacting with their colleagues in the classes. Other reasons stand, e.g., age variation in language classes, that the refugee students hold different age categories which creates extra challenges for their teachers and challenges in classmates interaction. A number of researchers (Casimiro et al., 2007; Riggs et al., 2012; Watkins et al., 2012) argued that when refugee students move to an overall new context, new scholastic environment, and start dealing with new teachers and new colleagues, this unsurprisingly creates many challenges, especially for foreign language learners. Ashton (1985) claimed that teachers’ self-efficacy affects refugee children’s academic success. In addition, Stevenson and Willott (2007) stated that the level of the family, homeland, and cultural and social background of refugees may stand as an obstruction in front of learning a foreign language. Olliff and Couch (2005) and Casimiro et al. (2007) argued that refugee language learners who did not experience official education in formal schools need more time and effort to be accustomed to learn a language. A study in Jordan by Alefesha and Al-Jamal (2019) found that refugees’ challenges in learning EFL are associated with poor educational background, discomfort with English, and lack of knowledge of teachers to deal with refugees. Conversely, research conducted by Riggs et al. (2012) concluded that refugees learn foreign languages better than peers due to two main reasons, namely, the first is to survive in the target country they live in, and the second is to get better job opportunities.

## Materials and Methods

### Data Collection Tool

An adapted questionnaire by Kurbegovic (2016) was employed to address teachers’ perceptions and challenges concerning teaching English to refugees, and some elements of the questionnaire were changed to meet the aim of the study.

The questionnaire was distributed online using Google forms, it consists of 6 categories (i.e., teachers’ self-efficacy, teachers’ practices, teachers’ cultural competency, teachers’ preparation, their refugees’ psychological needs, and refugee students’ linguistic challenges), and each article in each category has four alternative options (i.e., not at all, slightly agree, moderately agree, and greatly agree). The participants were not able to move to the questionnaire without confirming that they agree to participate in the study voluntarily.

### The Sample of the Study

The target population in the context of the study is large and it could not be covered by the researchers; still, a relatively small sample of the target population is satisfactory (Nworgu, 1991:69). Hence, the process of this research was applied to the accessible part of the population. The participants were 112 English language teachers who experience teaching English to refugee children in Jordan. The total number of responses to the questionnaire was 134, 22 responses were eliminated, and the selected responses stood at 112 ones.

### Validity and Reliability of the Instrument

The collected data were analyzed using SPSS25; the findings showed that the loadings of all the items related to students’ psychological needs, teachers’ self-efficacy, implementing practices, teachers’ cultural competencies, teachers’ preparation, and students’ linguistic challenges were more than 0.5 value; and the *p*-values were found to be statistically significant at less than 1% confidence level. This, according to some studies (Kock and Lynn, 2012; Adetola et al., 2021), is an indication that the measurement instrument used for the constructs demonstrates a good “convergent validity.” Furthermore, the “Cronbach’s alpha” and “composite reliability” coefficients for students’ needs (0.909 and 0.9304), students’ linguistic challenges (0.799 and 0.859), teachers’ preparation (0.847 and 0.894), teachers’ self-efficacy (0.919 and 0.936), implementing practices (0.933 and 0.946), and teachers’ cultural competency (0.774 and 0.843), respectively, were both higher than the conservative value of 0.7 (Kock, 2014, 2015), and this indicates that the measurement instrument has good reliability. The “average variance extracted” of students’ needs (0.690), students’ linguistic challenges (0.514), teachers’ preparation (0.636), teachers’ self-efficacy (0.675), implementing practices (0.715), and teachers’ cultural competency (0.50) are all greater than the threshold value of 0.5 (Kock, 2015; Adetola et al., 2021), which is an indication of an acceptable internal consistency. Finally, the associated “full collinearity variance inflation” (FVIF) with students’ needs (1.581), students’ linguistic challenges (1.404), teachers’ preparation (1.252), teachers’ self-efficacy (1.720), implementing practices (1.503), and teachers’ cultural competency (1.397) are all below the recommended threshold of less than (3.3).

## Results and Discussion

### English Language Teachers’ Preparation

Preparing and supporting teachers can equip them with the skills and knowledge to meet the needs of different students. Teachers need to take on action for career progress in the new merging situations, e.g., dealing with the waves of refugees in the classroom. In this study, 53% of English teachers do not agree on receiving sufficient in-service or preservice professional training on how best to support refugees in the classroom, or taking dedicated coursework in culturally responsive practices for refugees from diverse cultural backgrounds. Wasonga (2005) mentioned that teachers’ professional training supports them to feel prepared to meet the needs of their diverse students. De Jong and Harper (2005) recommended a special training for teachers of refugees and they stated that training for specific purposes for teachers, e.g., (refugee teaching) influences their efficacy.

### Self-Efficacy

Bandura (1986) defined self-efficacy as one’s capabilities to organize and achieve actions effectively. Teachers’ self-efficacy has been investigated in different areas, such as in math and science teaching (Tschannen-Moran and Hoy, 1998, p. 202). However, Klassen and Chiu (2011) claimed that research on teachers’ self-efficacy (TSE) in the contexts of foreign language teaching is still underrepresented within the literature. A high percentage (46%) of English teachers who participated in the study feel convinced that they can successfully teach English to refugee students which illustrates that they feel efficient in teaching English to refugees and this does not necessarily mean that they do not face any challenges, they also convinced that it is hard enough to exert a positive influence on both the personal and academic development of refugee students (Peeler and Jane, 2005), and they face difficulties maintaining a positive relationship with refugees during tensions.

### Cultural Competency

There is a huge increase in minority of students in schools due to the high number of refugees in host countries which made classes more diverse ethnically, culturally, and in terms of language and dialects. Many studies indicated that effective culturally responsive instruction, high-quality multicultural instruction, refugee students’ progress monitoring, and one-on-one learners’ support are critical for teaching diverse classes (Irvine, 2003; McIntyre, 2010). Being culturally illiterate can create serious threats to refugees’ academic progress. A high percentage (47.8%) of teachers greatly agreed that they are aware of the diversity in the classrooms due to the presence of refugees and they face challenges adapting methods to meet the culturally diverse students, and this instills high responsibility for teachers. Teachers agreed that they have not received enough training to meet culturally diverse students’ needs and this complies with a study by Goodwin (2002) who confirmed that teachers in different contexts experience many challenges while trying to meet the needs of culturally diverse students due to the typical preparation they receive from undergraduate degrees. According to McIntyre et al. (2010), language teacher education programs have to equip teachers of English to deliver instruction to various learners and prepare them to use methods that help refugees to achieve the literacy needed to progress in their education.

### Teachers’ Practices

Teachers play significant roles in providing quality education to refugee students. In this study, 60% of the participants agreed to have the willingness to implement new practices, such as cooperation with refugees’ families (Kirk and Winthrop, 2007) and cooperation between teachers and refugees’ families, which help refugees to restore a sense of stability and confidence. According to the analysis of teachers’ responses, they agreed on facing challenges with the practices they need to implement with refugees who suffer from mental and psychological problems. English teachers are not able to implement practices that meet refugee students’ needs, such as conducting or reading studies, trying new types of practices for refugee students by following a treatment manual, and using new and different types of practices developed by researchers. Although teachers can support refugees in the recovery phase in post-conflict emergencies, and with their practices they can promote security, safety, peace, and human rights upon return to home countries (Mononye, 2012), still this needs training. Crisp et al. (2001) argued that training teachers on implementing practices that help in meeting the diversity in the classroom can be challenging.

### Refugees’ Linguistic Challenges

Schools in Arab world countries teach different foreign languages, e.g., English is used as a foreign language in Jordan and Egypt, and French is used as a foreign language in Lebanon, Syria (some schools), and Morocco. Hence, some refugees learn French as a foreign language and after refuge, it becomes English, which created an obstacle to perform well academically (Riggs et al., 2012), and some of the refugees come with different foreign languages and they face linguistic challenges because of the unfamiliarity of the language taught in the new context; hence, English teachers recommend separation rather than inclusion. Moreover, the presence of refugees with different native languages poses a challenge for teachers and refugees. According to Cohen (2011), refugee students cannot communicate with their teachers due to the language barrier. The majority (60%) of the teachers greatly agreed that they suffer from refugees due to the lack of sufficient English proficiency which prevents refugees from meeting the basic academic requirements of school success. According to Fares et al. (2017), Syrian refugees suffer from learning a new language due to many reasons, such as curricula differences and sociocultural factors. They greatly agreed that teaching languages (English) is constructive (Piaget, 1980).

### Refugees’ Psychological Needs

Refugees’ psychological challenges have been investigated from teachers’ perspective, the results showed that the majority around 71% greatly agreed that refugees have unique emotional needs and seem more anxious or nervous compared to other students, and this affects their performance in language learning. Refugees show greater needs for emotional and behavioral support than peers (Forness et al., 2012), and this can be clarified by Krashen (1985) that the psychological status represented in anxiety and negative emotions prevents language acquisition or learning to occur effectively. He explained how psychological factors affect the foreign language learning process (Effective Filter Hypothesis).

Researchers found that refugees during wars experience traumatic events, and each experiences 7 and 15 traumatic events (Mollica et al., 1999). It is expected that this leaves a negative impact on the refugees, e.g., traumatic events lead to depression of 16% (Turner et al., 2003). In cognitive psychology studies, it has been found that traumatic experiences shift neural paths in the human brain and it affects the learning process. Individuals’ brain is designed to feel, receive, and store dangerous incidents; hence, all parts of the brain and body are involved in a “fight or flight response,” to enable individuals to respond to threatening situations. The traumatic events individuals pass through indeed impact the brain deeply and critically (Perry, 2006). The ability of processing information in individuals with post trauma stress disorder (PTSD) interferes with schema response, and the brain responds to the trigger with a pervasive terror response and initiates the fear response as if they were occurring for the first time. For learning new aspects of language, the brain needs to process, store, retrieve, and respond as it needs to be in a calm and attentive state. However, this flashback, or retriggering of fright response, places individuals’ brains in an extremely over-alert mode. This in turn prevents the progress of language learning process and offers promising relations for studies with traumatized language learners. According to Krashen (1985), anxiety and self-confidence are significant in second or foreign language acquisition; when self-confidence is low, it combines with debilitating anxiety and raises the learners’ affective filter, and then creates a block that declines comprehensible input to reach the language acquisition device.

### Model of Teacher–Refugee Relationship

To create this model, 5 hypotheses were tested, namely, H1: teachers’ preparation directly influences the practices they implement, H2: teachers’ preparation directly influences their self-efficacy, H3: teachers’ preparation directly influences their cultural competency, H4: practices that teachers implement influence (a) refugee students’ psychological needs and (b) refugee students’ linguistic challenges, H5: teachers’ self-efficacy influences (a) refugee students’ psychological needs and (b) refugee students’ linguistic challenges, and H6: teachers’ cultural competency influences (a) refugee students’ psychological needs and (b) refugee students’ linguistic challenges. To establish a relationship between these elements and represent them in a comprehensible model, IBM’s SPSS statistical tool has been employed to analyze the demographic characteristics of the respondents.

As the assessment of the measurement instrument reliability was examined, the discriminant validity of the constructs was measured as well (Table 1). The findings show conformity with the proposition in the literature that the “square root of average variance extracted shown in diagonal of each construct must be greater than the correlations between that construct and other constructs” (Fornell and Larcker, 1981). The result indicates that the students’ needs, students’ linguistic challenges, teachers’ preparation, teachers’ self-efficacy, implementing practices, and teachers’ cultural competency display good discriminant validity in the model context.

**TABLE 1:** Correlations among 1 vs. with square root of AVEs.

	SN	LC	*P*	SE	IP	CC
SN	**0.831**					
LC	0.461	**0.717**				
P	−0.229	−0.214	**0.797**			
SE	−0.140	−0.323	0.391	**0.822**		
IP	0.001	−0.211	0.309	0.542	**0.845**	
CC	0.381	0.078	0.065	0.296	**0.296**	**0.696**

*SN, students’ need; LC, students’ linguistic challenges; P, teachers’ preparation; SE, teachers’ self-efficacy; IP, implementing practices; CC, teachers’ cultural competency. Square roots of average variances extracted (AVEs) shown on diagonal. The numbers in bold indicate discriminant values.*

### Common Bias Method (CMB)

In respect to the “common method bias” (CMB), it was demonstrated in Kock’s (2015) study that the coefficients of “full collinearity VIF” are sensitive to “pathological common variations” across the constructs in methodological contexts that are the same with the one found in this study. Accordingly, it implies that the sensitivity enables CMB to be identified in a model which nevertheless passes the assessment of convergent and discriminant validity criteria based on a “confirmatory factor analysis” (CFA), as we have in this study. Some researchers suggested the value of 5 to be acceptable and < 3.3 to be the best for full collinearity VIF coefficients (Kock and Lynn, 2012; Kock, 2015; Adetola et al., 2021; Moguluwa et al., 2021). Thus, with the full VIF, none of the full VIF coefficients is greater than the acceptable threshold (≤5). Moreover, the “Stone-Geisser” (*Q*^2^) coefficients developed by Geisser (1974) and Stone (1974) are utilized for the assessment of predictive validity (Kock, 2015). This coefficient is only available for endogenous latent variables; that is those latent variables have arrows pointing at them. Kock (2015) suggested that a *Q*^2^ coefficient that is >0 indicates the acceptable predictive validity of the measurement model, and the results are presented in Table 2 and it shows that our model meets this criterion.

**TABLE 2:** Q-squared coefficients.

SN	LC	SE	IP	CC
0.288	0.229	0.160	0.123	0.049

*SN, students’ need; LC, students’ linguistic challenges; SE, teachers’ self-efficacy; IP, implementing practices; CC, teachers’ cultural competency.*

### Hypothesis Testing

For the structural model’s quality, the model fit indices were tested and then reported in Table 3. All the indices were either statistically significant or inconsistent with the respective thresholds, indicating that the quality of the structural model is adequate (Hair et al., 2010; Kock, 2020).

**TABLE 3:** Model fit and quality indices.

Indices	Coefficient	Decision
Average path coefficient (APC)	0.286	*P* < 0.001
Average R-squared (ARS)	0.163	*P* = 0.018
Average block VIF (AVIF)	1.154	Acceptable if ≤ 5, ideally ≤ 3.3
Average full collinearity VIF (AFVIF)	1.476	Acceptable if ≤ 5, ideally ≤ 3.3
Tenenhaus GOF (GOF)	0.318	Small ≥ 0.1, medium ≥ 0.25, large ≥ 0.36
R-squared contribution ration (RSCR)	1.000	Acceptable if ≥ 0.9, ideally = 1
Standardized root mean squared residual (SRMR)	0.070	Acceptable if ≤ 0.1

**FIGURE 1:**
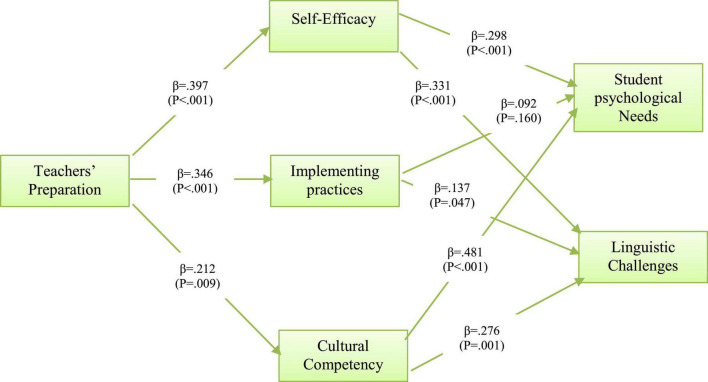
Model testing results.

Confirming the fitness of the model, the significance of the linear and non-linear relationships among the constructs was tested, and the *R* squared (*R*^2^) value as presented in Table 4 and Figure 1 in reference to Hair et al. (2019) indicates a moderate degree of variance explained in teachers’ self-efficacy (*R*^2^ = 0.16), implementing practices (*R*^2^ = 0.12), and low degree of variance explained in teachers’ cultural competency (*R*^2^ = 0.05) by teachers preparation. In addition, the result further shows that teachers’ self-efficacy, implementing practices, and teachers’ cultural competency contribute about 27 and 22% of explanation variation in students’ needs and students’ linguistic challenges, respectively.

**TABLE 4:** R-squared coefficient.

SN	LC	SE	IP	CC
0.275	0.219	0.158	0.120	0.045

*SN, students’ need; LC, students’ linguistic challenges; SE, teachers’ self-efficacy; IP, implementing practices; CC, teachers’ cultural competency.*

In accordance with the argument of Henseler et al. (2016) that the weight of the path coefficient should be examined through the evaluation of effect size (*f*^2^); Table 5 shows that teachers’ self-efficacy (0.058) and implementing practices (0.004) have a weak effect on students’ needs, while teachers’ cultural competency (0.213) has a strong effect on students’ needs which are following the recommendation of Cohen (1977). Similarly, self-efficacy (0.121), implementing practice (0.037), and teachers’ cultural competency (0.061) show a moderate, weak, and weak effect on student needs, respectively. In addition, the effect size as presented in Table 5 reveals that teachers’ preparation exerts a moderate effect on teachers’ self-efficacy and implementing practices while exerting a weak effect of teachers’ cultural competency.

**TABLE 5:** Effect size (*f*^2^).

Interaction	*f* ^2^
SE → SN	0.058
IP → SN	0.004
CC → SN	0.213
SE → LC	0.121
IP → LC	0.037
CC → LC	0.061
P → SE	0.158
P → IP	0.120
P → CC	0.045

*SN, students’ need; LC, students’ linguistic challenges; P, teachers’ preparation; SE, teachers’ self-efficacy; IP, implementing practices; CC, teachers’ cultural competency.*

The results of the model testing reveal the coefficients of the interaction of teachers’ preparation with teachers’ self-efficacy “H1” (β = 0.397, *p* < 0.001), implementing practices “H2” (β = 0.346, *p* < 0.001), and teachers’ cultural competency “H3” (β = 0.212, *p* = 0.009) to be positive and significant at 1% significance level, respectively. Therefore, hypotheses 1–3 were supported and concluded that teachers’ preparation has a significant influence on teachers’ self-efficacy, implementing practices, and teachers’ cultural competency. Moreover, the hypothesized direct relationship between teachers’ self-efficacy and students’ needs “H4a” (β = 0.298, *p* < 0.001) and between teachers’ self-efficacy and students’ linguistic challenges “H4b” (β = 0.331, *p* < 0.001) revealed to be positive and significant. This result indicated the significant impact of teachers’ self-efficacy on students’ needs and their linguistic challenges; hence, H4a and H4b were supported. The result of the estimating direct influence of implementing practices on students’ needs “H5a” (β = 0.092, *p* = 0.160) was found to be positive but not significant, while the coefficient of direct influence of implementing practices on students’ linguistic challenges “H5b” (β = 0.137, *p* = 0.047) as presented in Table 6 was found to be positive and significant. Thus, H5a was not supported, while H5b was supported; hence, we concluded that implementing practices directly influence the students’ linguistics challenge at less than 5% significance level. As for the direct influence of teachers’ cultural competency on both students’ needs “H6a” (β = 0.481, *p* < 0.001) and students’ linguistic challenges “H6b” (β = 0.276, *p* = 0.001), the results as presented in Table 6 revealed that teachers’ cultural competency exerts a positive influence on both students’ needs and their linguistic challenges. Therefore, H6a and H6b were supported.

**TABLE 6:** Path coefficients and *P* values.

Hypothesis	Interaction	Path coefficient (β)	*P*-value	Decision
H1	P → SE	0.397***	< 0.001	Supported
H2	P → IP	0.346***	< 0.001	Supported
H3	P → CC	0.212***	0.009	Supported
H4a	SE → SN	−0.298***	< 0.001	Supported
H4b	SE → LC	−0.331***	< 0.001	Supported
H5a	IP → SN	0.092	0.160	Not supported
H5b	IP → LC	−0.137**	0.047	Supported
H6a	CC → SN	0.481***	< 0.001	Supported
H6b	CC → LC	0.276***	0.001	Supported

SN, students’ need; LC, students’ linguistic challenges; P, teachers’ preparation; SE, teachers’ self-efficacy; IP, implementing practices; CC, teacher’ cultural competency. Values with * are significant at 10% level, values with ** are significant at 5% level, and values with *** are significant at 1% level.

The questionnaire data revealed significant correlations between teachers’ preparation and self-efficacy, teacher preparation and their practices, teacher preparation, and cultural competency. The results illustrated the importance of teacher preparation on teachers’ self-efficacy (Carr, 2013), practices (Cook et al., 2015) and cultural competency (Landa and Stephens, 2017). In addition, the data revealed significant correlations between self-efficacy and students’ psychological needs (Chwastek et al., 2021) and linguistic challenges (Alefesha and Al-Jamal, 2019), and between teachers’ practices and students’ psychological needs and linguistic challenges. Cultural competency with refugees’ psychological needs showed to be significant, and teachers’ cultural competency helps in meeting diverse students’ needs (Peeler and Jane, 2005; Lesaux and Geva, 2006). However, the correlation between teachers’ practices and students’ needs was not significant. As shown in the model, teachers’ self-efficacy, practices, and cultural competency mediate the relationship between teachers’ preparation and the linguistic challenges and psychological needs of refugee students. Hence, teacher preparation plays an important role in meeting refugee learners’ needs and challenges by preparing teachers adequately and effectively in terms of cultural competency, self-efficacy, and teachers’ practices.

## Conclusion

Despite the variation among refugee students, they share common challenges and experiences since they came from war-affected backgrounds. This study showed challenges associated with refugee learners of English and their teachers in Jordan, and attempted to find out the relationship between these challenges and present them in a model. The model showed that English teacher preparation influences teachers’ self-efficacy, cultural competency, and the practices teacher implement, and they influence refugees’ linguistic challenges and psychological needs. For example, according to the proposed model, if teachers had challenges with preparation, they would face challenges with the cultural competency and then this creates challenges in meeting refugees’ needs. The limitations of this study are associated with the phase of collecting data during the COVID-19 pandemic period. Future studies are likely to collect data from refugees qualitatively.

## Data Availability Statement

The raw data supporting the conclusions of this article will be made available by the authors, without undue reservation.

## Ethics Statement

The studies involving human participants were reviewed and approved by the Eastern Mediterranean University Ethics Committee. The patients/participants provided their written informed consent to participate in this study.

## Author Contributions

HA did the data collection, data analysis, wrote the introduction, methodology, and finally the discussion part of the manuscript. NK carried out the supervision of the manuscript. Both authors contributed to the article and approved the submitted version.

## Conflict of Interest

The authors declare that the research was conducted in the absence of any commercial or financial relationships that could be construed as a potential conflict of interest.

## Publisher’s Note

All claims expressed in this article are solely those of the authors and do not necessarily represent those of their affiliated organizations, or those of the publisher, the editors and the reviewers. Any product that may be evaluated in this article, or claim that may be made by its manufacturer, is not guaranteed or endorsed by the publisher.
