# Supplementary material for: Refugee Children and English Language: Challenges From English Language Teachers’ Perspectives
Source: Front Psychol. 2022 Jun 22;13:918734. doi: 10.3389/fpsyg.2022.918734 (PMC9257178; doi:10.3389/fpsyg.2022.918734)
Supplement: Supplementary file 1 [file Data_Sheet_1.pdf]

Measurement's properties assessment

Constructs	Loadings	Cronbach alpha	Composite reliability	Average variance extracted	FVIF
Students' Needs		.909	.930	.690	1.581
SN1	.846				
SN2	.816				
SN3	.876				
SN4	.733				
SN5	.868				
SN6	.838				
Students' Linguistic Challenges		.799	.859	.514	1.404
LC1	.853				
LC2	.818				
LC3	.564				
LC4	.826				
LC5	.682				
LC6	.472				
Teachers' Preparation		.847	.894	.636	1.252
P1	.838				
P2	.889				
P3	.506				
P4	.861				
P5	.830				
Teachers' Self-Efficacy		.919	.936	.675	1.720
SE1	.879				
SE2	.826				
SE3	.774				
SE4	.854				
SE5	.827				
SE6	.810				
SE7	.777				
Implementing Practices		.933	.946	.715	1.503
IP1	.844				
IP2	.876				
IP3	.848				
IP4	.895				
IP5	.855				

IP6	.818				
IP7	.777				
Teachers' Cultural Competence		.774	.843	.50	1.397
CC1	.738				
CC2	.788				
CC3	.753				
CC4	.788				
CC5*	.359				
CC6	.652				

Note: * denote excluded from final analysis

Path coefficients and P values

Hypothesis	Interaction	Path coefficient (β)	P value	Decision
H1	P → SE	.397***	<0.001	Supported
H2	P → IP	.346***	<0.001	Supported
H3	P → CC	.212***	0.009	Supported
H4a	SE → SN	-.298***	<0.001	Supported
H4b	SE → LC	-.331***	<0.001	Supported
H5a	IP → SN	.092	0.160	Not Supported
H5b	IP → LC	-.137**	0.047	Supported
H6a	CC → SN	.481***	<0.001	Supported
H6b	CC → LC	.276***	0.001	Supported

Note: SN = students' need, LC = students' linguistic challenges, P = teachers' preparation, SE = teachers' self-efficacy, IP = implementing practices, CC = teacher' cultural competency. * significant level at 10%, **5%, ***1%
